# The Experience of Women With Breast or Gynecological Cancer After Participation in an Online Mindfulness‐Based Cancer Recovery (e‐MBCR) Program: Secondary Outcomes Analysis of a Pilot Mixed Methods Randomized Controlled Trial

**DOI:** 10.1002/pon.70334

**Published:** 2025-11-21

**Authors:** Marie‐Estelle Gaignard, Dominica Martin, Jelena Stanic, Roger Hilfiker, Alexandre Bodmer, Michael Ljuslin, Khalil Zaman, Intidhar Labidi‐Galy, Apostolos Sarivalasis, Linda E. Carlson, Solange Peters, Pierre‐Yves Dietrich, Manuela Eicher, Guido Bondolfi, Françoise Jermann

**Affiliations:** ^1^ Department of Oncology Swiss Cancer Center Leman Geneva University Hospitals Geneva Switzerland; ^2^ Faculty of Medicine Center of Translational Research in Onco‐Hematology University of Geneva Geneva Switzerland; ^3^ Faculty of Psychology and Educational Sciences University of Geneva Geneva Switzerland; ^4^ Faculty of Biology and Medicine University of Lausanne and Lausanne University Hospital Institute of Higher Education and Research in Healthcare Lausanne Switzerland; ^5^ School of Health Sciences of the Canton of Vaud University of Applied Sciences and Arts Western Switzerland Lausanne Switzerland; ^6^ Division of Palliative Medicine Department of Rehabilitation and Geriatrics Geneva University Hospitals Geneva Switzerland; ^7^ Department of Oncology Swiss Cancer Center Leman Lausanne University Hospital Lausanne Switzerland; ^8^ Department of Oncology University of Calgary Calgary Alberta Canada; ^9^ Department of Psychiatry Geneva University Hospitals Geneva Switzerland; ^10^ Department of Psychiatry University of Geneva Geneva Switzerland

## Abstract

**Background:**

Mindfulness‐Based Interventions (MBIs) are recognized as beneficial in oncology supportive care. While qualitative analyses of mindfulness program experiences exist, this is the first mixed methods study to examine patients' experiences after participating in the online Mindfulness‐Based Cancer Recovery (e‐MBCR) program.

**Methods:**

The SERENITY study was a pilot randomized controlled trial evaluating the early implementation, and effects of the e‐MBCR program for women with breast or gynecological cancer in a French‐speaking context. This article reports on secondary outcomes from a mixed methods analysis, exploring psychosocial aspects through questionnaires and participants' experiences through interviews. Sixty‐two patients were randomized in a 2:1 ratio. Quantitative assessments were carried out at three timepoints; qualitative interviews only post‐intervention. Both datasets were analyzed separately, then merged for interpretation.

**Results:**

The intervention group showed a significant reduction in depression compared to the control group, with a medium effect size post‐intervention. While other psychological measures did not show significant differences, this exploratory analysis revealed favorable trends, particularly in anxiety, spiritual well‐being, and post‐traumatic growth. At 3‐month follow‐up, most scales showed a diminished effect compared to post‐intervention. Qualitative interviews revealed four themes: a safe and validating environment, acquiring skills and taking action, enhanced well‐being, and exposure to memories of cancer. This last dimension was a source of beneficial inner work for most participants, although it was a deeply challenging experience for four women.

**Conclusion:**

Participation in the e‐MBCR program led to beneficial experiences, notably self‐exploration, and enhanced self‐efficacy. However, MBIs can also bring up challenging experiences, which are important to acknowledge.

**Trial Registration:**

NCT04564768

**Abbreviations**
ASCO: American Society of Clinical Oncology
CHUV: Center Hospitalier Universitaire Vaudois (Lausanne University Hospital)
e‐MBCR: online Mindfulness‐Based Cancer Recovery
HUG: Hôpitaux Universitaires de Genève (Geneva University Hospitals)
MBCR: Mindfulness‐Based Cancer Recovery
MBI: Mindfulness‐Based Intervention
MBSR: Mindfulness‐Based Stress Reduction
SIO: Society for Integrative Oncology
TAU: Treatment As Usual

## 1 Background

Advances in cancer treatment resulting in increased rates and duration of survival have highlighted the importance of preserving patients' quality of life (QoL), which can be severely affected by the disease itself, and often by treatment side effects [1]. In this respect, supportive care has advanced considerably, in parallel with the rise of integrative oncology, that is the integration of complementary approaches alongside conventional care [2]. Without targeted psychosocial interventions, many patients experience stable or worsening trajectories in psychological outcomes such as anxiety, depression, fatigue, and QoL, often with persistently high unmet needs [3]. This underscores the need for supportive care approaches that actively promote coping and adjustment. Building on this, growing evidence has highlighted the value of Mindfulness‐Based Interventions (MBIs) [4, 5]. MBIs are a group of interventions adapted from the parent program Mindfulness‐Based Stress Reduction (MBSR), developed by Kabat‐Zinn in the late 1970s [6].

MBIs involve systematic training in mindfulness meditation, guided by a qualified professional to promote mental and physical health. They address most determinants of QoL in people living with cancer, with effect sizes ranging from small to large, and are particularly effective in reducing prevalent psychological distress, including anxiety and depression [7, 8, 9]. Inadequate recognition and management of these symptoms can decrease both QoL and survival [10]. In 2018, the American Society of Clinical Oncology (ASCO) endorsed Society for Integrative Oncology (SIO) recommendations advocating mindfulness‐based interventions to alleviate anxiety and depression and improve QoL of breast cancer survivors [4, 11]. To date, there are few studies on gynecological cancer patients [12], and even fewer on patients with metastatic disease [13].

The Mindfulness‐Based Cancer Recovery (MBCR) program was developed in the 2000s and adapted from the Mindfulness‐Based Stress Reduction (MBSR) program to meet the specific needs of people living with cancer [14]. It consists of 9 weekly group sessions and a 6‐h silent retreat, combining mindfulness practices and psycho‐educational exercises to better cope with stress and cancer‐related concerns [15].

While MBIs are effective and feasible for cancer patients, this does not guarantee successful implementation, highlighting the need to evaluate implementation processes [16]. Given the near absence of MBIs in oncology departments in the French‐speaking region of Switzerland, we conducted a pilot randomized controlled study evaluating the early implementation, as well as psychological and biological effects of the online MBCR program for women with breast or gynecological cancer (SERENITY study; NCT04564768) [17]. The online format was chosen because of the COVID‐19 pandemic, supported by emerging literature [18]. Here, we present secondary outcomes from a mixed methods analysis exploring the impact of the e‐MBCR program on psychological aspects via questionnaires and patients' experiences via interviews, offering deeper insight into their lived realities and the mechanisms supporting coping.

## 2 Method

### 2.1 Study Design

This pilot study followed a hybrid Type I [19], two‐armed randomized‐controlled design. The intervention group received the e‐MBCR program and treatment as usual (TAU), while the control group received TAU only and was placed on a waiting list to receive the mindfulness program after the end of the study.

The SERENITY study was conducted between 2020 and 2022 at two academic oncogynecological centers in the French‐speaking region of Switzerland (Geneva and Lausanne University Hospitals). A detailed flowchart of our study design has been published previously [17].

### 2.2 Participants

Participants were women with all stages of breast or gynecological cancer, after a minimum of 3 months from the end of their adjuvant treatment (for localized cancer) or without ongoing intravenous chemotherapy (for women with metastatic disease). Inclusion and exclusion criteria are described in detail in the study protocol [17].

### 2.3 Procedure

**FIGURE 1:**
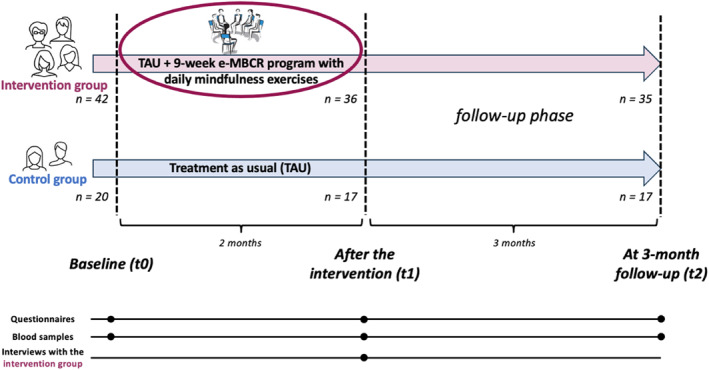
Study timeline and assessments.

Recruitment was carried out over 4 months before each program by oncologists and nurses. A psychologist interviewed the patients to assess the presence of any psychiatric exclusion criteria [17]. At baseline (t0), participants completed study questionnaires on the online secure REDCap platform. Sociodemographic and medical data were gathered. Participants were then randomly assigned (2:1) to intervention or control groups using a computer‐generated block randomization. Online questionnaires were completed after the intervention (t1), that is after 2.5 months, and at 3‐month follow‐up (t2), that is at around 6 months after t0. Additionally, semi‐structured interviews were conducted with the intervention group at t1. Figure 1 provides an overview of the study timeline and assessments.

### 2.4 The Intervention: E‐MBCR

The standardized 9‐week MBCR program incorporates various mindfulness practices such as meditation, body scan, and yoga, along with breathing and psycho‐educational exercises for coping with stress and cancer‐related concerns [15]. Participants are recommended to engage in a daily 45‐min home practice using guided audio meditations provided on a USB key. Additionally, they receive a booklet featuring didactic information, poems, and resources to continue practicing after the program.

The e‐MBCR program was delivered live through a secure institutional web platform. It was co‐facilitated by a certified and experienced MBSR teacher alongside an MBI‐certified psychiatric nurse, none of whom had previously led an MBCR program before. To ensure program's reliability, they were trained using the MBCR curriculum guide and other course materials.

### 2.5 Treatment as Usual (TAU) Wait‐List Control

TAU included routine follow‐up consultations with physicians and nurses. Patients with psychosocial needs could be referred to psycho‐oncological consultations. Control group participants were asked not to practice mindfulness during the study but were offered a free optional MBSR program afterward.

### 2.6 Outcome Measures

The assessment of secondary outcomes was conducted using psychometrically validated questionnaires and semi‐structured interviews. Details of all outcomes can be found in the study protocol [17]. They included scales measuring depression (MDI) [20] and anxiety (STAI) [21] symptoms, insomnia (SCI) [22], mindfulness (FFMQ) [23], quality of life (EORTC‐QLQ‐C30) [24], post‐traumatic growth (PTGI‐R) [25], spirituality (FACIT‐Sp12) [26], self‐compassion (SCS) [27] and fear of cancer recurrence (FCRI) [28]. Participants completed questionnaires on the secure REDCap platform, taking about 45 min. At each visit, information on engagement with complementary therapies was collected. The intervention group also participated in a semi‐structured interview at t1, discussing their experience with the e‐MBCR program (as part of a broader interview that covered study procedures and other implementation processes). The central question for the analysis presented here was: *What was your experience with the mindfulness program you participated in?*

### 2.7 Analysis

The sample size of 72 participants was determined based on the primary feasibility outcome, that is adherence to e‐MBCR sessions, and was not powered to assess efficacy. Exploring psychological effects and participants' experiences with the program was part of secondary outcomes. We summarized baseline characteristics using descriptive statistics. These were compared between groups using *t*‐tests or chi‐square analyses to ensure randomization success. All analyses were performed following a modified intention‐to‐treat (ITT) approach, using all participants with follow‐up data for at least one time point, under an assumption of missing at random. For the mixed method analysis, quantitative and qualitative data were analyzed separately, then merged for interpretation in the discussion, according to a Triangulation Design [29].

Mean scores and confidence intervals for all measures of secondary outcomes were estimated for each group at baseline, t1, and t2. Comparisons of scores across these time points between groups were performed using mixed models with a random intercept for each participant, using the restricted maximum likelihood (REML) method. The Kenward‐Roger method was used for computing the degrees of freedom, with an autoregressive structure of order 1 for the residual errors, adjusting for baseline values of the outcome variables. Dependent variables were standardized to interpret the intervention effect as a Cohen's d effect size between groups, with a positive effect indicating improvement in the intervention group. Cohen's d effect sizes were estimated using the dppc2 formula, and Phi (*φ*) was used for Chi‐square tests, both interpreted using Cohen's standards; a d value of approximately 0.2 indicating a small effect, 0.5 a medium effect, and 0.8 or higher a large effect. Missing data for each outcome was documented.

The qualitative analysis was conducted by the first co‐authors (DM, MEG), using *Braun & Clarke* thematic approach [30]. The data set consisted of 35 interviews, of which 24 were fully transcribed, after which data saturation was achieved. The remaining 11 recordings were listened to, paraphrased, and coded, with no new codes emerging. Codes were generated inductively then reviewed, refined, and grouped into sub‐themes and themes based on their occurrence (at the interview level). To ensure trustworthiness, we used investigator triangulation, team discussions and reflexive debriefings. While member checking was not performed, themes were closely grounded in participants' verbatim accounts. MAXQDA software was used to organize quotes, codes, and themes throughout the analysis.

## 3 Results

### 3.1 Participants' Characteristics

While the target sample size was 72, 62 women were included—42 in the intervention group and 20 in the control group—primarily due to recruitment challenges during the COVID‐19 pandemic. Although we aimed to recruit patients with gynecological cancer, they were underrepresented, likely due to recruitment by oncologists rather than gynecologists, and the exclusion of those undergoing intravenous chemotherapy. Their characteristics are described in Table 1. Due to dropouts, 53 participants (36 intervention, 17 control) were included in the post‐intervention analysis, and 52 (35 intervention, 17 control) at 3‐month follow‐up. Of the six participants who dropped out of the intervention group, three withdrew at baseline, opting not to begin the e‐MBCR program. The primary reasons for the withdrawals in the intervention group were organizational constraints and illness‐related factors. A detailed CONSORT flow diagram and comprehensive information on attrition will be reported in a separate implementation‐focused publication.

**TABLE 1:** Participants' characteristics.

	Intervention	Control	Total
*N*	42.0 (67.7%)	20.0 (32.3%)	62.0 (100.0%)
Age. Median [IQR]	49.0 [44.0–60.0]	56.0 [46.5–63.0]	52.0 [45.0–61.0]
Years since cancer diagnosis. Median [IQR]	3.0 [1.0–6.0]	2.0 [1.0–6.5]	2.5 [1.0–6.0]
Highest education			
Primary school	1.0 (2.8%)	1.0 (6.2%)	2.0 (3.8%)
Secondary school	3.0 (8.3%)	4.0 (25.0%)	7.0 (13.5%)
Maturity/Bac	9.0 (25.0%)	3.0 (18.8%)	12.0 (23.1%)
University	21.0 (58.3%)	6.0 (37.5%)	27.0 (51.9%)
Apprenticeship/CFC	2.0 (5.6%)	2.0 (12.5%)	4.0 (7.7%)
Cancer type			
Gynecologic	4.0 (9.5%)	3.0 (15.0%)	7.0 (11.3%)
Breast	38.0 (90.5%)	17.0 (85.0%)	55.0 (88.7%)
Cancer stage			
0	1.0 (2.4%)	0.0 (0.0%)	1.0 (1.6%)
I	7.0 (16.7%)	5.0 (25.0%)	12.0 (19.4%)
II	10.0 (23.8%)	9.0 (45.0%)	19.0 (30.6%)
III	11.0 (26.2%)	3.0 (15.0%)	14.0 (22.6%)
IV	13.0 (31.0%)	3.0 (15.0%)	16.0 (25.8%)
Localized patients: Currently under endocrine therapy			
No	8.0 (27.6%)	7.0 (41.2%)	15.0 (32.6%)
Yes	21.0 (72.4%)	10.0 (58.8%)	31.0 (67.4%)
Localized patients: Currently under anti‐HER2			
No	27.0 (93.1%)	14.0 (82.4%)	41.0 (89.1%)
Yes	2.0 (6.9%)	3.0 (17.6%)	5.0 (10.9%)
Metastatic patients: Currently under endocrine therapy (incl. CDK4/6 inhibitors)			
No	9.0 (69.2%)	3.0 (100.0%)	12.0 (75.0%)
Yes	4.0 (30.8%)	0.0 (0.0%)	4.0 (25.0%)
Metastatic patients: Currently under p.o. ChT, anti‐HER2 or PARPi			
No	6.0 (46.2%)	0.0 (0.0%)	6.0 (37.5%)
Yes	7.0 (53.8%)	3.0 (100.0%)	10.0 (62.5%)

Abbreviation: IQR: Interquartile range, that is 25 and 75 percentile.

### 3.2 Psychological Outcomes

**FIGURE 2:**
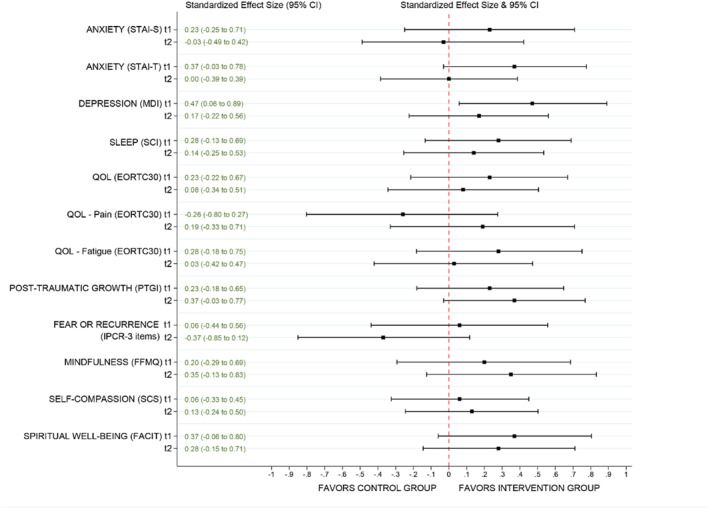
Caterpillarplot representing the effect sizes after the intervention (t1) and at 3‐month follow‐up (t2) by mixed methods analysis.

The mixed model findings of the Time × Condition analysis are presented in Table 2. Given the small sample size of this feasibility study, these statistical comparisons should be considered exploratory. We observed a statistically significant decrease in the depression score (MDI) with a medium effect size (d: 0.47, *p* = 0.03) in the intervention group after the intervention (t1) compared to the control group. A score below 25 indicates mild depressive symptomatology [20]. For the rest of the scales, all effect sizes were in favor of the intervention, except for pain, although these did not reach statistical significance. Figure 2 summarizes the effect sizes at t1 and t2. Notably, Trait anxiety (STAI‐T) (d: 0.37, *p* = 0.07) and spiritual well‐being (FACIT) (d: 0.37, *p* = 0.09) showed medium effect sizes at t1. At 3‐month follow‐up (t2), there was a clear reduction in effect on all scales, except for the post‐traumatic growth (PTGI) scale, which showed an improvement at t2 with a statistically non‐significant medium effect size (d: 0.37, *p =* 0.07). For descriptive purposes, within‐group effect sizes are reported in Table S1.

**TABLE 2:** Mean (SD) per group and time‐point, as well as the between group differences in absolute and standardized mean differences.

t0			t1					t2				
Outcomes	Intervention	Control	Intervention	Control	Between group	Effect size	*p*‐value	Intervention	Control	Between group	Effect size	*p*‐value
	Mean (SD)	Mean (SD)	Mean (SD)	Mean (SD)	Between group difference			Mean (SD)	Mean (SD)	Between group difference		
Anxiety (STAI‐S)	40 (14.1)	33.4 (7.6)	34.5 (34.5)	33.4 (10.7)	2.52 (−2.87 to 7.9)	0.23	0.359	37 (12.7)	31.1 (7.8)	−3.75 (−10.58 to 3.09)	−0.31	0.283
Anxiety (STAI‐t)	40.1 (12.2)	33.6 (8)	36.2 (36.2)	34.2 (9.3)	3.57 (−0.39 to 7.53)	0.37	0.077	36.3 (9.7)	30.5 (7.7)	−2.89 (−8.83 to 3.05)	−0.28	0.34
Depression (MDI)	14.7 (9.3)	8.5 (4.1)	11 (11)	9.5 (6.2)	3.28 (0.11 to 6.44)	0.47	0.043	11.8 (7.2)	8.5 (6.8)	−1.68 (−6.34 to 2.97)	−0.22	0.478
Sleep (SCI)	17 (7.5)	22.1 (7.7)	20.6 (20.6)	22.7 (6.2)	1.89 (−1.03 to 4.81)	0.28	0.205	20.5 (7.1)	23.1 (6.8)	−1.69 (−5.87 to 2.48)	−0.23	0.426
QOL (EORTC30)	64.6 (19.1)	75 (17.8)	69.7 (69.7)	73.9 (22)	5.08 (−4.64 to 14.79)	0.23	0.306	69.3 (21.3)	76.7 (13.4)	−6.03 (−19.14 to 7.07)	−0.29	0.367
QOL ‐ pain (EORTC30)	29.6 (27.1)	24.4 (20.8)	26.4 (26.4)	16.7 (19.9)	−5.53 (−18.65 to 7.6)	−0.26	0.409	26.7 (25)	25.6 (24.3)	3.62 (−10.45 to 17.7)	0.14	0.614
QOL ‐ fatigue (EORTC30)	42.6 (27.8)	29.6 (24.4)	34 (34)	31.1 (25.6)	7.41 (−4.8 to 19.62)	0.28	0.234	37.1 (28)	26.7 (17.7)	−6.9 (−22.39 to 8.58)	−0.26	0.382
Post‐traumatic growth (PTGI)	63.1 (20)	41.5 (23.3)	68.2 (68.2)	44.7 (26.4)	5.9 (−4.37 to 16.18)	0.23	0.26	71 (19.7)	44 (25)	22.62 (9.34 to 35.91)	0.94	0.001
Fear or recurrence (IPCR‐3 items)	1.5 (1.5)	2.8 (2.7)	1.1 (1.1)	2.2 (2.8)	0.11 (−0.89 to 1.11)	0.06	0.827	1.3 (2)	1.6 (2.3)	0.02 (−1.13 to 1.17)	0.01	0.973
Mindfulness (FFMQ)	131 (18.6)	139.7 (15.5)	140.6 (140.6)	143.9 (17.7)	3.64 (−5.38 to 12.66)	0.20	0.429	143.4 (18.6)	142.9 (12.8)	2.34 (−8.72 to 13.41)	0.13	0.678
Self‐compassion (SCS)	3.3 (0.7)	3.6 (0.6)	3.6 (3.6)	3.8 (0.7)	0.05 (−0.22 to 0.32)	0.06	0.741	3.6 (0.7)	3.8 (0.7)	−0.14 (−0.55 to 0.27)	−0.2	0.495
Spiritual well‐being (FACIT)	29.6 (8.6)	31.5 (8.1)	33.2 (33.2)	32 (8.8)	2.78 (−0.52 to 6.08)	0.37	0.098	33 (7.8)	32.6 (6.9)	1.85 (−2.62 to 6.32)	0.24	0.417

### 3.3 Qualitative Results

The data set comprised 35 interviews, ranging from 16 to 63 min (*m* = 41, SD = 14). When asked about their overall experience of the e‐MBCR program, participants reported four key themes: (a) A safe and validating environment, (b) Acquiring skills and taking action, (c) Enhanced well‐being, and (d) Exposure to memories of cancer. All themes and codes are summarized in Table 3.

**TABLE 3:** Summary of themes, sub‐themes and codes.

Themes	Sub‐themes & codes	*N*
A safe and validating environment [32/91%]	Qualities of the instructors Human qualities [24], knowledge and expertise [21].	30 (86%)
	Social connectedness Being in a group [22], a group of women with cancer [20].	28 (80%)
Acquiring skills and taking action [32/91%]	Core mindfulness skills Accepting more and reacting/judging less [17], listening/observing myself [17], being conscious of my own body [14], better controlling my attention [8].	28 (80%)
	Coping strategies Coping with stress and difficult feelings [21], self‐compassion/self‐kindness [14], changing perspective [10], expressing my needs [7].	25 (71%)
	Sense of self‐efficacy Giving myself self‐care [13], using mindfulness in everyday life [6], taking care of my well‐being and global health [5].	17 (49%)
Enhanced well‐being [31/89%]	At psychological level Less stress and more serenity [21], less irritability/anger [11], better mood [7], better concentration [8].	27 (77%)
	At body level Improved perception of bodily sensations [17], better sleep [11], relaxation [9], greater mind‐body connection [9], less pain [9], greater mobility [6].	25 (71%)
	In my relationships with others	13 (37%)
	In my life with cancer	8 (23%)
Exposure to cancer memories [23/66%]	A context reminiscent of cancer (due to) Group of patients [16], attention toward the body [7], content of the booklet/practices [5], by participating in the program [3], thoughtfulness of instructors [2].	21 (60%)
	Beneficial inner work Outcomes: Hope and personal growth [6], rebuilding oneself [6], less fear of recurrence [6], kindness toward oneself/others/the universe [4], focusing on life [3]. Process: Moving past denial and avoidance [9], meditating exposes to thoughts [6], becoming aware/putting words to it [6].	14 (40%) 14 (40%) 10 (29%)
	Difficult experiences Triggers: Practices as cues to cancer [2], others ‘recurrence or sufferings [2], silent day [2]. Strategies: Denial [4], avoidance [4]. Symptoms: Pain [2], fear of recurrence [2], identity of ill person [2].	4 (11%) 4 (11%) 4 (11%) 4 (11%)

*Note: N* total = 35 participants.

#### 3.3.1 A Safe and Validating Environment

Most participants described the context provided by the e‐MBCR program as safe, supportive, and validating of their identity and cancer experience. Teachers were portrayed as mindfulness experts and their human qualities were highlighted:

I found them really wonderful, very attentive, patient and they always had the right words when we intervened, to reassure us, to put our minds at rest. They really understood what we were feeling, even if we couldn't express it very well. Really, an extraordinary kindness.

Two participants, however, felt that the teachers' attentiveness—especially during yoga—reinforced a sense of being seen as “fragile”.

The social connectedness within the group was frequently cited as crucial. Being among women with cancer provided peer support, reduced feelings of isolation, and fostered belonging—described as “a small cocoon” or “my little Serenity family.” This group dynamic also shifted their focus from illness and offered an inspiring environment:

Seeing women in remission for ten years is very helpful.

#### 3.3.2 Acquiring Skills and Taking Action

Most participants highlighted learning new skills, grouped as “core mindfulness skills” and “coping strategies”. Core mindfulness skills included being less reactive and judgmental, more accepting, and more aware of their experiences and actions. They said they had discovered a new way of connecting with themselves. Body awareness, especially through body scans, and improved attention regulation were also reported.

Over two‐thirds of patients reported learning coping strategies, helping them manage stress and difficult emotions. Participants also experienced increased self‐compassion and kindness toward themselves. Finally, they mentioned gaining perspective and managing relationships better by expressing their needs more clearly.

Before the program, I used to say, “No, my priority is to keep the house running.” And when I started meditating, I learned to say, “No, I come first”.

These skills seem to have given half of the participants a sense of self‐efficacy in managing their lives and health, particularly in facing fear of recurrence and cancer‐related ruminations:

I didn't see how a mindfulness program could help people with any illness, cancer or otherwise, and I was wrong. Because yes, illness isn't going to go away with meditation. But well‐being is a global thing, from head to toe. It's not just a question of healthy or cancerous cells, and that is what I also learned, which is to take complete responsibility for myself.

#### 3.3.3 Enhanced Well‐Being

Most participants reported improved well‐being, whether psychological, physical, interpersonal, or in their overall life with cancer. Benefits included reduced stress, increased serenity, and less irritability. Participants noted that the program helped calm their minds, acting like a “switch” or “booster” to improve their mindset. Additionally, some noticed clearer thinking, better concentration, and a shift from “automatic mode” to a greater appreciation of “gifted time”.

More than two‐thirds experienced physical benefits such as an improvement in awareness of bodily sensations, relaxation, feeling lighter, and breathing more freely. Many attributed these changes specifically to the practices of mindful breathing and body scan. A third of participants mentioned improved sleep:

I used to have restless sleep; when I woke up, it didn't feel like I had slept; meditation brought me this calm during the day, and at night as well.

A few participants observed a change in their relationship to pain, with mindful yoga helping them recognize and manage pain better. Additionally, some reported improved mobility from practicing yoga.

Just over a third described enhanced relationships—with more desire to engage, fewer family conflicts, and improved work dynamics, stating that “being well with oneself contributes to better interactions with others”.

#### 3.3.4 Exposure to Cancer Memories

A fourth theme that surfaced for two‐thirds of participants was that of exposure to their memories of cancer. Despite no direct questions about the disease, cancer‐related experiences emerged strongly during the interviews. Most participants suggested that the program's hospital setting and the interactions with other cancer patients created a context reminiscent of their own cancer journey.

During these sessions, even if we didn’t always talk about the disease, it reminded us why we were there.

A few patients reported that yoga and body scan practices heightened their awareness of bodily issues related to their cancer, prompting them to reconnect with parts of their body they had distanced themselves from to cope with the disease. Meditations and poems referencing illness also served as reminders of their condition.

For 40 percent of all participants, exposure to difficult memories led to beneficial inner work, including personal and spiritual growth. They reported “rebuilding their lives”, developing greater self‐kindness, and experiencing less fear of recurrence, along with a deeper appreciation of the present moment.

The worries are still there because the disease is still there. We hope it never comes back but the fear is always there. This program taught me to see and accept that fear, and that there are ways to manage and maybe accept it. It doesn’t weigh on me anymore.

Some patients suggested that these changes were due to mindfulness helping them move past denial or avoidance, allowing them to confront their thoughts, become more aware of their experiences, and articulate their feelings more effectively.

It is important to mention some undesirable effects that were discussed during the interviews; four participants, including three who left the program midway through, experienced increased psychological distress. They reported that the group sessions, the day of silence, or hearing about another participant's relapse conflicted with their strategy of keeping the disease or at bay. It also led to more pain, as well as a resurgent sense of illness proximity and increased fear of recurrence:

I don’t even think about the disease every day. When I go to the doctor, yes, I think, “I’m sick, I need to see a doctor,” but otherwise, no. It's as if I've locked it away in a little box in my head. But every Friday, I had to open that box, and it was beginning to weigh heavily on me.

## 4 Discussion

This work aimed to present secondary outcomes of the SERENITY study, that is the psychological outcomes and experiences of participants with breast or gynecological cancer following their participation in the e‐MBCR program. Quantitative results showed a statistically significant decrease in depressive symptoms after the intervention, particularly relevant in this population. Even modest improvements can be clinically meaningful, given that these symptoms are associated with a risk of major depression [31]. There was no statistically significant effect on the other scales, which is not surprising for this feasibility pilot trial with a small sample size, where patients were not recruited based on psychological distress. As such, these findings should be considered exploratory, especially since effect sizes must be interpreted cautiously in the absence of significant group × time interactions. While our models accounted for initial scores, effect sizes are reported as descriptive indicators of change rather than as evidence of intervention efficacy. Nonetheless, favorable trends in anxiety (the Trait subscale in particular), spiritual well‐being, and post‐traumatic growth scale at 3‐month follow‐up were consistent with prior research [5, 32, 33, 34]. The pain difference favoring the control group remained unexplained, despite review of complementary care and medication use.

Combining quantitative and qualitative analyses provided a deeper understanding of the e‐MBCR experience. Qualitative data confirmed the improvement in psychological well‐being, including reduced stress and greater serenity, while highlighting the skills gained and the beneficial inner work resulting from the program. These findings also supported quantitative data on post‐traumatic growth, with participants noting acceptance, greater self‐compassion, and shifts in perspective. The post‐traumatic growth questionnaire showed that these effects persisted and increased at the 3‐month follow‐up. Although “spiritual well‐being” didn't explicitly emerge, themes such as greater serenity or body connection suggested a concordance with improved elements of the FACIT‐Sp questionnaire. Besides, some of the sub‐themes in the “Beneficial inner work” category included the notion of meaning in life, central to the definition of spirituality as understood in contemporary healthcare [35].

While the direct benefits of mindfulness are well‐documented, qualitative findings emphasized the significant role of skills acquired through participation in an MBI. According to Hölzel's model [36], these skills include core mindfulness skills like focused attention and acceptance, along with self‐regulation skills that help manage ruminations and worries, collectively alleviating depressive and anxiety symptoms [36, 37]. Our study confirmed that these skills enhance coping abilities and perceived self‐efficacy, aligning with a recent systematic review [38]. This being particularly relevant, as high self‐efficacy leads to empowerment, which in turn reinforces self‐efficacy in everyday situations [39]. Implementing MBIs in oncology could thus be framed not just as learning meditation, but as a tool for self‐knowledge and regulation.

The program's learning environment was also critical. A strong sense of group support and community emerged, even online, reflecting known factors in self‐exploration and personal growth [40]. Additionally, although MBIs are not commonly considered a psychotherapy setting, the concept of therapeutic alliance [40, 41]—where participants felt heard, understood, and emotionally connected with their teachers—emerged as a key factor. This alliance, combined with peer support and skills acquisition, provided participants with optimal conditions for personal growth. These findings suggest that implementing MBIs in oncology should not only focus on delivering therapeutic content, but also actively promote a supportive group dynamic and therapeutic alliance.

Finally, this study, through its qualitative analysis, sheds light on the inner work induced by e‐MBCR. It seems that it exposed participants to cancer‐related memories through mindfulness practices, interactions with fellow patients, and specific psycho‐educational content. Although this re‐exposure supported beneficial inner work for many participants, it was profoundly challenging for four individuals. While confronting distressing thoughts can aid coping [38], trauma theory cautions that such re‐exposure without proper processing may cause re‐traumatization [42]. Therefore, as some authors suggest [43], it is important to teach participants to stay within a “window of tolerance” in order to manage difficult emotions safely.

This raises the question of whether participants were sufficiently informed to cope with these potential side effects. Notably, these four participants were in the same group. Two of them reported a distressing experience during the introductory session, when another patient spoke of the recurrence of her metastatic cancer. These participants, who were in remission and said they hadn't expected to be confronted with their cancer fears again, found this re‐exposure emotionally unsettling. Nonetheless, the challenging experiences were transient, and despite not utilizing the psycho‐oncology consultation offered, patients reported feeling better quickly after discontinuing the program. These findings evoke the concept of “set and setting,” central in psychedelic‐assisted therapy [44], emphasizing how participants' mindsets and the intervention setting affect outcomes. This highlights the need to provide comprehensive information (e.g., on the “window of tolerance”), set clear expectations, and ensure teachers are adequately trained to create a safe and supportive environment [45]. Experienced facilitators are generally able to help participants process and work through difficult emotions and memories that often emerge in the first few weeks of the program.

### 4.1 Clinical Implications

In addition to the well‐documented psychological and physical benefits of mindfulness, structured MBIs offer valuable skills for people living with cancer, such as acceptance, perspective‐shifting, and self‐compassion, which are likely to be maintained over time. Since MBIs may expose participants to difficult cancer‐related memories, it is essential to offer these programs with caution by qualified and experienced teachers, preferably trained in oncology according to ASCO guidelines [11]. Pre‐program individual interviews—common in MBIs—are also critical for understanding patients' needs, clarifying expectations, and informing them about potential challenges and coping strategies.

### 4.2 Study Limitations

This study has several limitations, primarily its small sample size and use of an inactive control group. The 2:1 randomization, chosen to increase exposure to the intervention in this pilot context, resulted in a small control group, potentially further reducing statistical power and group comparability. Moreover, not selecting patients based on psychological distress likely contributed to the low statistical significance. Another limitation is the underrepresentation of gynecological cancer patients, which limited subgroup‐specific conclusions. On the qualitative side, all intervention participants were interviewed, including women with both early and metastatic cancer, making the sample representative. However, the broad interview question may have limited the exploration of themes less spontaneously addressed by patients, but which could nonetheless be important determinants of their QoL.

### 4.3 Future Research

Given the study's findings, it is important to further investigate the psychological mechanisms behind the inner work experienced by participants and how it aids in overcoming cancer‐related challenges. Research should address how therapeutic processes can be optimized while ensuring adequate support for patients who may encounter difficult experiences, particularly in this population facing ongoing stressors, even years after their diagnosis. Recent research on trauma‐sensitive mindfulness is relevant here [46]. Additionally, exploring mindfulness's impact on patients' spirituality could offer valuable insights into their QoL, an aspect still too often neglected in cancer care.

## 5 Conclusion

This mixed methods study found that women with breast or gynecological cancer had a beneficial experience with the e‐MBCR program. The program provided a safe and validating context, improving psychological and physical well‐being as well as the learning of skills promoting self‐efficacy and beneficial inner work. However, participation also brought up cancer‐related memories—an experience that proved beneficial for most, but challenging for some. These aspects should be considered when implementing MBIs in oncology, ensuring a thorough understanding of patients' psychological states, clarifying expectations, and informing them about the potential challenges of mindfulness practice and how to address them.

## Author Contributions

Conceptualization, Writing – review and editing, and approval of the submitted manuscript: All co‐authors. Methodology, Investigation: Marie‐Estelle Gaignard, Dominica Martin, Jelena Stanic, Roger Hilfiker, Alexandre Bodmer, Michael Ljuslin, Linda E. Carlson, Manuela Eicher, Guido Bondolfi, Françoise Jermann. Formal analysis: Marie‐Estelle Gaignard, Dominica Martin, Jelena Stanic, Roger Hilfiker, Françoise Jermann. Supervision: Linda E. Carlson, Manuela Eicher, Guido Bondolfi, Françoise Jermann. Writing – original draft: Marie‐Estelle Gaignard, Dominica Martin. Funding acquisition: Marie‐Estelle Gaignard, Manuela Eicher, Guido Bondolfi.

## Funding

This study was supported by the Research and Development Grant from Geneva University Hospital, the Swiss Cancer Research Foundation, the Leenaards Foundation, and the NOVA Foundation.

## Ethics Statement

The SERENITY study (2019‐00965) was approved by the Cantonal Ethics Committees of Geneva and Vaud and conducted in accordance with the principles of the Declaration of Helsinki.

## Consent

Written informed consent was obtained from all study participants prior to enrollment.

## Conflicts of Interest

The authors declare no conflicts of interest.

## Supporting information


**Table S1:** Paired within‐group effect sizes (Cohen's d) for control and intervention groups across timepoints.

## Data Availability

The datasets generated and/or analyzed during the current study are available from the corresponding author on reasonable request.

## References

[1] A. J. Biparva , S. Raoofi , S. Rafiei , et al., “Global Quality of Life in Breast Cancer: Systematic Review and Meta‐Analysis,” BMJ Supportive & Palliative Care 13, no. e3 (December 2023): e528–e536, 10.1136/bmjspcare-2022-003642. PMC10850719 35710706

[2] C. M. Witt , L. G. Balneaves , M. J. Cardoso , et al., “A Comprehensive Definition for Integrative Oncology,” Journal of the National Cancer Institute Monographs 2017, no. 52 (November 2017), 10.1093/jncimonographs/lgx012. 29140493

[3] M. A. Franzoi , A. Di Meglio , S. Michiels , et al., “Patient‐Reported Quality of Life 6 Years After Breast Cancer,” JAMA Network Open 7, no. 2 (February 2024): e240688, 10.1001/jamanetworkopen.2024.0688. 38421653 PMC10905303

[4] G. H. Lyman , H. Greenlee , K. Bohlke , et al., “Integrative Therapies During and After Breast Cancer Treatment: ASCO Endorsement of the SIO Clinical Practice Guideline,” Journal of Clinical Oncology 36, no. 25 (September 2018): 2647–2655, 10.1200/jco.2018.79.2721. 29889605 PMC13123314

[5] N. G. Xunlin , Y. Lau , and P. Klainin‐Yobas , “The Effectiveness of Mindfulness‐Based Interventions Among Cancer Patients and Survivors: A Systematic Review and meta‐analysis,” Supportive Care in Cancer 28, no. 4 (April 2020): 1563–1578, 10.1007/s00520-019-05219-9. 31834518

[6] J. Kabat‐Zinn , “University of Massachusetts Medical Center/Worcester, Stress Reduction Clinic,” in Full Catastrophe Living: Using the Wisdom of Your Body and Mind to Face Stress, Pain, and Illness (Delacorte Press, 1990).

[7] H. Haller , M. M. Winkler , P. Klose , G. Dobos , S. Kümmel , and H. Cramer , “Mindfulness‐Based Interventions for Women With Breast Cancer: An Updated Systematic Review and Meta‐Analysis,”Acta Oncologica 56, no. 12 (December 2017): 1665–1676, 10.1080/0284186x.2017.1342862. 28686520

[8] M. P. Shiyko , S. Hallinan , and T. Naito , “Effects of Mindfulness Training on Posttraumatic Growth: A Systematic Review and Meta‐Analysis,” Mindfulness 8, no. 4 (2017): 848–858, 10.1007/s12671-017-0684-3.

[9] L. E. Carlson , A. Waller , S. L. Groff , J. Giese‐Davis , and B. D. Bultz , “What Goes Up Does Not Always Come Down: Patterns of Distress, Physical and Psychosocial Morbidity in People With Cancer Over a One Year Period,” Psycho‐Oncology 22, no. 1 (January 2013): 168–176, 10.1002/pon.2068. 21971977

[10] A. Pitman , S. Suleman , N. Hyde , and A. Hodgkiss , “Depression and Anxiety in Patients With Cancer,” BMJ 361 (April 2018): k1415, 10.1136/bmj.k1415. 29695476

[11] L. E. Carlson , N. Ismaila , E. L. Addington , et al., “Integrative Oncology Care of Symptoms of Anxiety and Depression in Adults With Cancer: Society for Integrative Oncology–ASCO Guideline,” Journal of Clinical Oncology 41, no. 28 (October 2023): 4562–4591, 10.1200/jco.23.00857. 37582238

[12] L. Stafford , N. Thomas , E. Foley , et al., “Comparison of the Acceptability and Benefits of Two Mindfulness‐Based Interventions in Women With Breast or Gynecologic Cancer: A Pilot Study,” Supportive Care in Cancer 23, no. 4 (April 2015): 1063–1071, 10.1007/s00520-014-2442-6. 25281227

[13] A. Kubo , E. Kurtovich , M. McGinnis , et al., “Pilot Pragmatic Randomized Trial of Mhealth Mindfulness‐Based Intervention for Advanced Cancer Patients and Their Informal Caregivers,” Psycho‐Oncology 33, no. 2 (February 2024): e5557, 10.1002/pon.5557. 32979294

[14] L. Carlson and M. Speca , Mindfulness‐Based Cancer Recovery: A Step‐by‐Step MBSR Approach to Help You Cope With Treatment and Reclaim Your Life (New Harbinger Publications, 2011), 210.

[15] L. E. Carlson , E. Zelinski , K. Toivonen , and M. Flynn , “Mindfulness‐Based Cancer Recovery: An Adaptation of MBSR for People With Cancer and Their Caregivers,” in Handbook of Mindfulness‐based Programmes (Routledge, 2019).

[16] M. S. Bauer , L. Damschroder , H. Hagedorn , J. Smith , and A. M. Kilbourne , “An Introduction to Implementation Science for the Non‐Specialist,” BMC Psychology 3, no. 1 (September 2015): 32, 10.1186/s40359-015-0089-9. 26376626 PMC4573926

[17] Gaignard M. E. , J. Stanic , A Bodmer , et al., “The SERENITY Study: Online Mindfulness‐Based Cancer Recovery (e‐MBCR) Program for Women Living With Breast and Gynecological Cancer—Protocol for a Pilot Effectiveness‐Implementation Randomized Trial,” Journal of Psychosocial Oncology Research and Practice [Internet]. 5, no. 2 (June 2023): [cited 2024 Nov 29], https://journals.lww.com/jporp/fulltext/2023/04000/the_serenity_study__online_mindfulness_based.2.aspx.

[18] K. A. Zernicke , T. S. Campbell , M. Speca , K. McCabe‐Ruff , S. Flowers , and L. E. Carlson , “A Randomized Wait‐List Controlled Trial of Feasibility and Efficacy of an Online Mindfulness‐based Cancer Recovery Program: The Etherapy for Cancer Applying Mindfulness Trial,” Psychosomatic Medicine 76, no. 4 (May 2014): 257–267, 10.1097/psy.0000000000000053. 24804884

[19] N. Pearson , P. J. Naylor , M. C. Ashe , M. Fernandez , S. L. Yoong , and L. Wolfenden , “Guidance for Conducting Feasibility and Pilot Studies for Implementation Trials,” Pilot and Feasibility Studies 6, no. 1 (October 2020): 167, 10.1186/s40814-020-00634-w. 33292770 PMC7603668

[20] P. Bech , N. A. Rasmussen , L. R. Olsen , V. Noerholm , and W. Abildgaard , “The Sensitivity and Specificity of the Major Depression Inventory, Using the Present State Examination as the Index of Diagnostic Validity,” Journal of Affective Disorders 66, no. 2–3 (October 2001): 159–164, 10.1016/s0165-0327(00)00309-8. 11578668

[21] Spielberger C. D. , R. L. Gorsuch , P. R. Vagg , and G. A. Jacobs , Manual for the State‐Trait Anxiety Inventory (Self‐evaluation Questionnaire) [Internet]. (Consulting Psychologists Press, 1983): [cited 2024 Dec 16], https://cir.nii.ac.jp/crid/1370285712575158016.

[22] C. A. Espie , S. D. Kyle , P. Hames , M. Gardani , L. Fleming , and J. Cape , “The Sleep Condition Indicator: A Clinical Screening Tool to Evaluate Insomnia Disorder,” BMJ Open 4, no. 3 (March 2014): e004183, 10.1136/bmjopen-2013-004183. PMC3964344 24643168

[23] R. A. Baer , G. T. Smith , E. Lykins , et al., “Construct Validity of the Five Facet Mindfulness Questionnaire in Meditating and Nonmeditating Samples,” Assessment 15, no. 3 (September 2008): 329–342, 10.1177/1073191107313003. 18310597

[24] N. K. Aaronson , S. Ahmedzai , B. Bergman , et al., “The European Organization for Research and Treatment of Cancer QLQ‐C30: A Quality‐of‐Life Instrument for Use in International Clinical Trials in Oncology,” Journal of the National Cancer Institute 85, no. 5 (March 1993): 365–376, 10.1093/jnci/85.5.365. 8433390

[25] R. G. Tedeschi and L. G. Calhoun , “The Posttraumatic Growth Inventory: Measuring the Positive Legacy of Trauma,” Journal of Traumatic Stress 9, no. 3 (July 1996): 455–471, 10.1007/bf02103658. 8827649

[26] A. R. Munoz , J. M. Salsman , K. Stein , and D. Cella , “Reference Values of the Functional Assessment of Chronic Illness Therapy – Spiritual Well‐Being (FACIT‐Sp‐12): A Report From the American Cancer Society’s Studies of Cancer Survivors,” Cancer 121, no. 11 (June 2015): 1838–1844, 10.1002/cncr.29286. 25712603 PMC4441564

[27] K. D. Neff , “The Development and Validation of a Scale to Measure Self‐Compassion,” Self and Identity 2, no. 3 (2003): 223–250, 10.1080/15298860309027.

[28] S. Simard and J. Savard , “Fear of Cancer Recurrence Inventory: Development and Initial Validation of a Multidimensional Measure of Fear of Cancer Recurrence,” Supportive Care in Cancer 17, no. 3 (March 2009): 241–251, 10.1007/s00520-008-0444-y. 18414902

[29] J. W. Creswell and V. L. Plano Clark , Designing and Conducting Mixed Methods Research (SAGE Publications, 2007), 275.

[30] V. Braun and V. Clarke , “Using Thematic Analysis in Psychology,” Qualitative Research in Psychology 3, no. 2 (2006): 77–101, 10.1191/1478088706qp063oa.

[31] R. Zhang , X. Peng , X. Song , et al., “The Prevalence and Risk of Developing Major Depression Among Individuals With Subthreshold Depression in the General Population,” Psychological Medicine 53, no. 8 (2023): 3611–3620, 10.1017/s0033291722000241. 35156595 PMC10277767

[32] C. H. Oner , B. Bayir , S. Sayar , and M. Demirtas , “Effect of Mindfulness‐Based Therapy on Spiritual Well‐Being in Breast Cancer Patients: A Randomized Controlled Study,” Supportive Care in Cancer 31, no. 7 (July 2023): 438, 10.1007/s00520-023-07904-2 37395841

[33] L. E. Carlson , R. Tamagawa , J. Stephen , E. Drysdale , L. Zhong , and M. Speca , “Randomized‐Controlled Trial of Mindfulness‐based Cancer Recovery Versus Supportive Expressive Group Therapy Among Distressed Breast Cancer Survivors (MINDSET): Long‐term Follow‐Up Results,” Psycho‐Oncology 25, no. 7 (July 2016): 750–759, 10.1002/pon.4150. 27193737

[34] F. Faghani , A. Choobforoushzadeh , M. R. Sharbafchi , and H. Poursheikhali , “Effectiveness of Mindfulness‐Based Supportive Psychotherapy on Posttraumatic Growth, Resilience, and Self‐Compassion in Cancer Patients : A Pilot Study,” Wiener Klinische Wochenschrift 134, no. 15–16 (August 2022): 593–601, 10.1007/s00508-022-02057-4. 35849181

[35] de Brito Sena M. A. , R. F. Damiano , G. Lucchetti , and M. F. P. Peres , “Defining Spirituality in Healthcare: A Systematic Review and Conceptual Framework,” Frontiers in Psychology [Internet] 12 (November 2021): 756080: [cited 2024 Nov 29], https://www.frontiersin.org/journals/psychology/articles/10.3389/fpsyg.2021.756080/full. 34867654 10.3389/fpsyg.2021.756080 PMC8637184

[36] B. K. Hölzel , S. W. Lazar , T. Gard , Z. Schuman‐Olivier , D. R. Vago , and U. Ott , “How Does Mindfulness Meditation Work? Proposing Mechanisms of Action From a Conceptual and Neural Perspective,” Perspectives on Psychological Science 6, no. 6 (November 2011): 537–559, 10.1177/1745691611419671. 26168376

[37] J. Gu , C. Strauss , R. Bond , and K. Cavanagh , “How Do Mindfulness‐Based Cognitive Therapy and Mindfulness‐Based Stress Reduction Improve Mental Health and Wellbeing? A Systematic Review and Meta‐Analysis of Mediation Studies,” Clinical Psychology Review 37 (April 2015): 1–12, 10.1016/j.cpr.2015.01.006. 25689576

[38] J. M. Heinen , E. M. Laing , N. Schäffeler , et al., “How Do Mindfulness‐Based Interventions Promote Coping and self‐efficacy in Patients With Cancer: A Systematic Review of Qualitative and Quantitative Data,” Psycho‐Oncology 33, no. 5 (2024): e6350, 10.1002/pon.6350. 38777617

[39] E. Rawlett Kristen , Journey from Self‐Efficacy to Empowerment [Internet] (Sciknow Publications Ltd. Health Care, 2014): [cited 2024 Nov 29], https://www.researchgate.net/publication/260184054_Journey_from_Self‐Efficacy_to_Empowerment.

[40] M. J. Lambert and D. E. Barley , “Research Summary on the Therapeutic Relationship and Psychotherapy Outcome,” Psychotherapy: Theory, Research, Practice, Training 38, no. 4 (2001): 357–361, 10.1037/0033-3204.38.4.357.

[41] E. S. Bordin , “The Generalizability of the Psychoanalytic Concept of the Working Alliance,” Psychotherapy Theory Research and Practice 16, no. 3 (1979): 252–260, 10.1037/h0085885.

[42] S. L. Shapiro , L. E. Carlson , J. A. Astin , and B. Freedman , “Mechanisms of Mindfulness,” Journal of Clinical Psychology 62, no. 3 (March 2006): 373–386, 10.1002/jclp.20237. 16385481

[43] D. J. Siegel , The Developing Mind: How Relationships and the Brain Interact to Shape Who We Are, 3rd ed. (Guilford Press, 2020), 674.

[44] I. Hartogsohn , “Constructing Drug Effects: A History of Set and Setting,” Drug Science, Policy and Law 3 (January 2017): 2050324516683325, 10.1177/2050324516683325.

[45] E. Shonin and W. Van Gordon , “Practical Recommendations for Teaching Mindfulness Effectively,” Mindfulness 6, no. 4 (August 2015): 952–955, 10.1007/s12671-014-0342-y.

[46] D. R. Garfin , A. Amador , J. Osorio , K. S. Ruivivar , A. Torres , and A. M. Nyamathi , “Adaptation of a Mindfulness‐Based Intervention for Trauma‐Exposed, Unhoused Women With Substance Use Disorder,” Psychological Trauma: Theory, Research, Practice, and Policy (June 2023), 10.1037/tra0001486 PMC12102743 37307346

