# Supplementary material for: The Experience of Women With Breast or Gynecological Cancer After Participation in an Online Mindfulness‐Based Cancer Recovery (e‐MBCR) Program: Secondary Outcomes Analysis of a Pilot Mixed Methods Randomized Controlled Trial
Source: Psychooncology. 2025 Nov 21;34(11):e70334. doi: 10.1002/pon.70334 (PMC12639194; doi:10.1002/pon.70334)
Supplement: Supplementary file 1 — Table S1: Paired within‐group effect sizes (Cohen's d) for control and intervention groups across timepoints. [file PON-34-e70334-s001.docx]

**Supplementary Table S1.** Paired within-group effect sizes (Cohen’s d) for control and intervention groups across timepoints.

| **Outcomes** | **ES t1-t0 (Contr)** | **ES t2-t1 (Contr)** | **ES t2-t0 (Contr)** | **ES t1-t0 (Inter)** | **ES t2-t1 (Inter)** | **ES t2-t0 (Inter)** |
| --- | --- | --- | --- | --- | --- | --- |
| ANXIETY (STAI-S) | 0.00 | 0.24 | 0.19 | 0.46 | -0.30 | 0.23 |
| ANXIETY (STAI-T) | -0.09 | 0.50 | 0.25 | 0.58 | -0.11 | 0.43 |
| DEPRESSION (MDI) | -0.25 | 0.23 | -0.06 | 0.71 | -0.27 | 0.35 |
| SLEEP (SCI) | 0.13 | 0.12 | 0.18 | 0.74 | -0.08 | 0.51 |
| QOL (EORTC30) | -0.08 | 0.19 | 0.04 | 0.27 | -0.13 | 0.22 |
| QOL - Pain (EORTC30) | 0.34 | -0.50 | -0.20 | 0.14 | -0.02 | 0.03 |
| QOL - Fatigue (EORTC30) | -0.07 | 0.20 | -0.03 | 0.46 | -0.21 | 0.17 |
| POST-TRAUMATIC GROWTH (PTGI) | 0.18 | 0.05 | 0.09 | 0.40 | 0.11 | 0.59 |
| FEAR OR RECURRENCE (IPCR-3 items) | 0.34 | 0.45 | 0.77 | 0.22 | -0.13 | 0.10 |
| MINDFULNESS (FFMQ) | 0.51 | -0.11 | 0.29 | 0.51 | 0.14 | 0.63 |
| SELF-COMPASSION (SCS) | 0.53 | 0.14 | 0.42 | 0.54 | 0.05 | 0.68 |
| SPIRITUAL WELL-BEING (FACIT) | 0.09 | 0.09 | 0.01 | 0.64 | -0.05 | 0.49 |
